# Quality of Life and Cochlear Implant: Results in Saudi Children

**DOI:** 10.7759/cureus.11968

**Published:** 2020-12-08

**Authors:** Ola Alnuhayer, Yazeed Alshawi, Bedoor Julaidan, Norah Alromaih, Norah Alakeel, Abdulaziz Alballaa

**Affiliations:** 1 College of Medicine, King Saud University, Riyadh, SAU; 2 Otolaryngology, Prince Sultan Military Medical City, Riyadh, SAU; 3 Otolaryngology, King Saud University, Riyadh, SAU

**Keywords:** cochlear implants (ci), quality of life (qol), pedsql™ 4.0 - gcs, toddlers, young children

## Abstract

Objective

To assess the quality of life (QoL) in Saudi children with cochlear implants (CI) and determine sociodemographic and clinical factors that have an impact on the perceived QoL.

Methods

A cross-sectional study is performed by comparing the QoL of Saudi toddlers and young CI recipients with normal children, using the Paediatric Quality of Life Inventory 4.0 - Generic core scale (PedsQL^TM^ 4.0 - GCS). A self-administered questionnaire was sent to parents of paediatric patients, who had cochlear implantation at King Abdulaziz University Hospital (KAUH), from March 2016 to March 2018. Mothers of age-matched normal children who attended the obstetrics and gynaecology clinics at King Khalid University Hospital (KKUH), in November 2019 were considered as a control group.

Results

When children with CI and normal children PedsQL^TM^ 4.0 - GCS subscales (physical, emotional, social, and school) and total functioning scores were compared, no single significant difference was noted between the groups. The sociodemographic and clinical factors that have an impact on the perceived QoL were: gender, birth order, and distance from the CI center. Emotional, social, psychosocial, and total functioning were the main dimensions affected.

Conclusion

The QoL of Saudi children with CI is comparable to those of normal children. However, among children with CI, gender, birth order, and distance from the CI center were found to have different effects on the QoL dimensions.

## Introduction

Hearing impairment has a significant impact on the lives of patients, their families, and society as a whole. According to the World Health Organization (WHO), it is estimated that disabling hearing loss affects around 466 million people worldwide, considering hearing loss as the most frequent sensory impairment. It is also predicted that the number of people with disabling hearing loss will rise in the upcoming years. Around 7% of the total people affected with hearing loss are children (34 million) of which 0.9% (1.4 million) are living in the Middle East and North Africa [1]. A study was carried out in Riyadh, in which 2574 Saudi preschool children aged between 4 and 8 years were included. The outcomes reflected that the prevalence of hearing loss was estimated to be 1.75% of which 15.6% have sensorineural hearing loss [2]. Childhood-onset hearing impairment can have a significant impact on the social and academic performances of the children, affecting their speech and language domains [3]. The importance of early exposure to a spoken language lies in the development and evolution of social and cognitive skills and both are tightly associated with language abilities [4]. Delayed language development in children with hearing impairment can also have emotional and behavioural problems, and all of these factors can influence the household stress level as stated by Quittner et al. [5] and accordingly, necessitate intervention in such children. Several studies have been done to demonstrate the benefits of cochlear implants (CI) on children, who are affected with profound hearing loss. These studies evaluated a variety of outcomes, including speech perception, auditory perception, hearing, receptive language, expressive language, communication, social functioning, academic functioning, and quality of life (QoL) [6].

QoL is one of the outcomes assessed to measure the success of cochlear implantation. WHO defines the QoL as, “an individual's perception of their position in life in the context of the culture and value systems in which they live and in relation to their goals, expectations, standards, and concerns.” QoL reflects a complex interaction between the individual’s physical health, mental health, social health, functional health, and personal characteristics, and thus, individualizes the meaning of QoL among different persons [7,8]. According to the definition of QoL, it is suitable for the individuals (including children) to self-report and assesses their QoL; however, we can refer to the proxy to measure the QoL especially in children with disabilities [9,10]. The Paediatric Quality of Life Inventory 4.0 - Generic core scale (PedsQL^TM^ 4.0 - GCS) is a reliable and valid paediatric health-related QoL instrument that can be used in both healthy children and children with different health conditions. In a study that was done in Kuwait, they concluded that the Arabic version of PedsQL^TM^ 4.0 - GCS is understandable, feasible, and can be used to evaluate paediatric health outcomes in research [11]. Due to the lack of any study that measures QoL in children with CI in Saudi Arabia, the aim of our study is to assess the QoL in toddlers and young children with CI and to look for sociodemographic and clinical factors that impact the perceived QoL. By doing this, we can understand the patients' experiences with the CI, its effects in both the medical and psychophysical aspects, and possible opportunities for helping children with CI to live and enjoy a productive satisfactory life.

## Materials and methods

Study design

Between September 2019 and November 2019, a cross-sectional case-control study was performed to assess the QoL of toddlers and young children with CI compared to normal children and determine if sociodemographic or clinical factors have an influence on the perceived QoL among toddlers and young children with CI.

Participants/study population

The participants were paediatric patients who had cochlear implantation at King Abdullah Ear Specialist Centre (KAESC) at King Abdulaziz University Hospital (KAUH), King Saud University, from March 2016 to March 2018. The criterion for inclusion was as follows: (1) all paediatric patients with CI with ages between 2 and 7 years. The exclusion criteria were: (1) children with other medical or psychological problems (either acute or chronic); (2) children with cognitive impairment; (3) any patient whose questionnaire was filled-up with someone other than the direct caregiver. The control participants were mothers of age-matched normal children who attended obstetrics and gynaecology clinics (antenatal, maternal medicine, and general gynaecology clinics) at King Khalid University Hospital (KKUH), King Saud University, in November 2019.

Data collection methods

Google Form was used to build our questionnaire. In the questionnaire, we used the validated Arabic version of PedsQL^TM^ 4.0 - GCS, parent proxy-report form. Permission to use the Arabic (Saudi Arabia) validated version of this instrument was granted by Mapi Trust (the owner of the PedsQL site) [12]. PedsQL^TM^ 4.0 - GCS has many forms for different age groups. In our study, we were targeting two age groups; toddlers (ages 2 - 4) and young children (ages 5 - 7). PedsQL^TM^ 4.0 - GCS (parent proxy-report form) is composed of 21 items for toddlers and 23 items for young children. These items are divided into four main dimensions which include the physical, emotional, social, and school functioning of the child during the previous four weeks. For each item, there are five responses [0 = never, 1 = almost never, 2 = sometimes, 3 = often, 4 = almost always]. Items will be reverse-scored and linearly transformed to a 0-100 scale as follows: 0 =100, 1= 75, 2 = 50, 3 =25, 4 = 0, so that higher scores indicate better health-related QoL. We calculated the mean score for each dimension with the summation of the items over the number of items answered in each dimension and we calculated the total score with the summation of all the items over the number of items answered on all the scales [13]. In the second part of the questionnaire, there were eight questions about the sociodemographic characteristics of the participants. These include age, gender, a region of Saudi Arabia, mother's and father's educational levels, family monthly household income, number of siblings, and birth order in the family. In the end, for children with CI, there were additional four questions about their experiences with CI. These include the duration of cochlear implantation, whether the child had experienced complications related to surgery, whether the child had experienced technical problems related to CI, and compliance to speech rehabilitation after cochlear implantation. Details of the child's clinical condition, including age at diagnosis, age of cochlear implantation, and IQ were extracted from patient records.

Sample size and sampling technique

The calculated sample size for our study was 134 with 67 children with CI and 67 normal children in order to detect the difference between two independent means with a detection power of 0.95. A sample size of 54 toddlers and young children with CI was found to be enough to perform correlation analysis with a detection power of 0.95. The sample size was calculated by a software program (G*Power, version 3.1.9.4) recommended by Bonett and Wright [14]. The means and the standard deviations were obtained from a previous study [15]. We took all toddlers and young children with CI who meet the inclusion/exclusion criteria. In the control group, systemic randomization was done. Every second mother who attended obstetrics and gynaecology clinics in November 2019 and has an age-matched normal child was included.

Data analysis plan

Data were analysed using SPSS Pc + 21.0 version statistical software (IBM Corp., Armonk, NY). Descriptive statistics such as frequencies, percentages, means, and standard deviations were used for describing the categorical and quantitative variables. Statistical tests like Pearson’s chi-squared test, independent sample t-test, ANOVA, Pearson correlation coefficient, were used to perform the correlation analysis. A p-value of ≤ 0.05 and a 95% confidence interval were used to report the statistical significance and precision of results.

Ethical considerations

The study was conducted after the approval of the King Saud University review board with approval number E-19-4135. All participants were informed about the purpose of the study and were given a written electronic informed consent, which was printed on the first page of the survey. The study ensured that the participant’s data will be confidential, private, and only used for research purposes.

## Results

A total of 147 participants were included in our study. For the CI group, 73 children were recruited and 74 normal children were recruited as controls. Data of participant demographics are displayed in Table 1. The clinical and social details for paediatric patients with CI are displayed in Table 2.

**Table 1:** The sociodemographic characteristics of paediatric patients with CI and normal controls

Characteristics	Children with CI	Normal children
	n (%)	n (%)
Total number	73	74
Age		
2	1 (1.4%)	7 (9.5%)
3	13 (17.8%)	14 (18.9%)
4	22 (30.1%)	14 (18.9%)
5	21 (28.8%)	19 (25.7%)
6	10 (13.7%)	12 (16.2%)
7	6 (8.2%)	8 (10.8%)
Gender		
Male	48 (65.8%)	33 (44.6%)
Female	25 (34.2%)	41 (55.4%)
Mother’s educational level		
Less than high school graduate	9 (12.3%)	6 (8.1%)
High school graduate	15 (20.5%)	19 (25.7%)
Diploma or equivalent	9 (12.3%)	4 (5.4%)
Bachelor’s degree	38 (52.1%)	37 (50%)
Higher education	2 (2.7%)	8 (10.8%)
Father’s educational level		
Less than high school graduate	9 (12.3%)	1 (1.4%)
High school graduate	19 (26%)	13 (17.6%)
Diploma or equivalent	11 (15.1%)	8 (10.8%)
Bachelor’s degree	32 (43.8%)	32 (43.2%)
Higher education	2 (2.7%)	20 (27%)
Family monthly household income (Saudi Riyals)		
<5000	8 (11%)	7 (9.5%)
5000–9999	32 (43.8%)	18 (24.3%)
10,000–14,999	20 (27.4%)	30 (40.5%)
15,000–19,999	10 (13.7%)	11 (14.9%)
≥20,000	3 (4.1%)	8 (10.8%)

**Table 2:** The clinical and social factors of paediatric patients with CI ^(1)^Complications related to surgery such as wound infection, dizziness/vertigo, headache, loss of appetite. ^(2)^Technical problems related to CI such as wire damage, rapid consumption of battery, strange sounds, inability to hear soft/loud sounds.

Variable	n (%) or Mean (± SD)
Age (in months)	57.49 (± 14.61)
Age at diagnosis (in months)	14.56 (± 11.67)
Age at cochlear implantation (in months)	31.97 (± 14.75)
Duration since CI (in months)	24.45 (± 11.27)
IQ	94.41 (± 9.59)
Experienced complications related to surgery ^(1)^	
Yes	7 (9.6 %)
No	66 (90.4 %)
Experienced technical problems related to CI ^(2)^	
Yes	8 (11 %)
No	65 (89 %)
Compliance to speech rehabilitation	
Yes	47 (64.4 %)
No	26 (35.6 %)
Number of siblings	
None	4 (5.5 %)
[1-3]	46 (63 %)
[4-6]	14 (19.2 %)
More than 6	9 (12.3 %)
Birth order in the family	
First-born	18 (24.7 %)
Middle-born	20 (27.4 %)
Last-born	35 (47.9 %)
Region	
Central region	41 (56.2 %)
Eastern region	6 (8.2 %)
Western region	5 (6.8 %)
Northern region	4 (5.5 %)
Southern region	17 (23.3 %)
Distance from CI centre	
Less than 200 km	37 (50.7 %)
More than 200 km	36 (49.3 %)

Participants characteristics

Pearson's chi-squared test was initially conducted to determine the similarity of the two participant groups on relevant key variables - age, gender, mother's and father's educational levels, family monthly household income, and parental marital status. Only gender and father’s education level were significantly different between the groups. Males comprised 65.8% of CI group and 44.6% of normal group (p = 0.01). The father’s education level within the normal group was significantly higher than the CI group (p = 0.0001), showing 70.2% have a bachelor’s degree and higher education compared to only 46.5% among the CI group. However, when comparing the family monthly household income, there was no significant difference (p = 0.081).

Comparison of children with CI to normal children reports for the PedsQL - GCS

The PedsQL^TM ^4.0 - GCS subscales (physical, emotional, social, school and psychosocial) and total score means are provided in Table 3. The mean of the total PedsQL score for the children with CI was 87.08 (±11.10), whereas the mean score for the control group was 88.57 (±11.30). When the children with CI and normal children GCS subscales and total functioning score means were compared using independent samples t-tests, no single significant difference was noted between the groups.

**Table 3:** The PedsQL - GCS scores of paediatric patients with CI and normal controls

PedsQL - GCS scores by domain	Children with CI	Normal children	p-Value
	Mean (±SD)	Mean (±SD)	
Physical PedsQL score	92.97 (±10.70)	92.145 (±12.09)	0.662
Emotional PedsQL score	87.26 (±15.50)	83.11 (±15.10)	0.102
Social PedsQL score	87.33 (±15.05)	87.77 (±16.20)	0.864
School PedsQL score	80.72 (±16.82)	83.87 (±15.60)	0.355
Psychosocial PedsQL score	84.33 (±13.66)	85.62 (±12.87)	0.554
Total PedsQL score	87.08 (±11.10)	88.57 (±11.3)	0.423

Association of the studied factors with PedsQL - GCS subscales and total scores among children with CI

ANOVA test, independent sample T-test, and Pearson correlation coefficient were used to assess the association and relationship between the PedsQL - GCS subscales and total scores with sociodemographic and clinical factors. The results are summarized in Tables 4 and 5.

**Table 4:** The association of the studied factors with PedsQL - GCS subscales and total scores among children with CI

Characteristics	Physical PedsQL	Emotional PedsQL	Social PedsQL	School PedsQL	Psychosocial PedsQL	Total PedsQL
	Mean (±SD)	Mean (±SD)	Mean (±SD)	Mean (±SD)	Mean (±SD)	Mean (±SD)
Gender						
Male	92.38 (±11.4)	84.48 (±15.9)	85.1 (±15.3)	80.12 (±16.2)	81.98 (±13.5)	85.46 (±10.9)
Female	94.1 (±9.4)	92.6 (±13.4)	91.6 (±13.9)	82.19 (±17.7)	88.83 (±13)	90.21 (±10.9)
p-value	0.52	0.033	0.08	0.684	0.041	0.082
Mother’s educational level						
Less than high school graduate	98.26 (± 5.2)	90.56 (±14.2)	96.11 (±6.9)	80.71 (±20.5)	90.53 (±11.1)	93.01 (±9)
High school graduate	94.99 (±10.3)	88.00 (±14.1)	90.00 (±16.2)	79.85 (±16.5)	84.75 (±13.8)	88.24 (±10.4)
Diploma or equivalent	89.58 (±12.5)	90.56 (±17)	92.22 (±9.7)	89.52 (±16.3)	86.15 (±14.9)	87.01 (±12.9)
Bachelor’s degree	91.69 (±11.3)	84.74 (±16.3)	82.37 (±15.7)	77.79 (±16)	81.43 (±13.6)	84.59 (±11)
Higher education	93.43 (±9.3)	100	100	100	100	99.45 (±0.8)
p-value	0.39	0.53	0.035	0.24	0.17	0.13
Father’s educational level						
Less than high school graduate	95.48 (±9.2)	88.33 (±12.5)	95.56 (±9.2)	76.66 (±16.1)	88.58 (±11.2)	90.86 (±10)
High school graduate	95.06 (±10.4)	90.79 (±11.8)	90 (±11.5)	83.59 (±18)	85.68 (±11.2)	88.88 (±8.9)
Diploma or equivalent	88.92 (±10.9)	81.36 (±15.8)	85.91 (±16.2)	76.43 (±14.2)	82.15 (±15.2)	84.86 (±12.1)
Bachelor’s degree	92.38 (±11.4)	86.09 (±17.9)	84.06 (±17.1)	80.38 (±17.6)	82.51 (±15.3)	85.21 (±12.2)
Higher education	92.97 (±10.7)	100	85.00 (±21.2)	95.83 (±5.9)	93.26 (±9.5)	95.28 (±5.1)
p-value	0.59	0.39	0.30	0.59	0.61	0.42
Family monthly household income (Saudi Riyals)						
<5000	89.06 (±13)	81.25 (±12.7)	88.75 (±8.7)	74.16 (±12.3)	80.92 (±9.1)	83.68 (±9.1)
5000–9999	90.6 (±12.3)	89.38 (±14.8)	87.81 (±14.4)	79.62 (±18.5)	83.54 (±14.7)	85.39 (±12.5)
10,000–14,999	95.47 (±8.7)	84.5 (±18.7)	83 (±19.5)	79.33 (±17.9)	82.34 (±15.6)	86.93 (±11.2)
15,000–19,999	97.81 (± 4.2)	88 (±13.9)	90.5 (±11.6)	92.07 (±12.5)	90.6 (±8.5)	93.13 (±5.7)
≥20,000	95.83 (±7.2)	96.67 (±5.8)	96.67 (±5.8)	84.16 (±1.2)	94.1 (±1.5)	95.14 (±3.2)
p-value	0.20	0.48	0.51	0.28	0.33	0.19
Birth order in the family						
First-born	91.29 (±12.1)	76.07 (±19.5)	81.07 (±19.6)	74.7 (±20.5)	75.04 (±16.9)	80.08 (±13.9)
Middle-born	95.9 (±8.5)	92.25 (±11.2)	90 (±14.05)	81.11 (±17.9)	88.47 (±10.7)	90.45 (±9.3)
Last-born	92.05 (±11.7)	88.29 (±14.5)	88 (±14.05)	83.47 (±14.7)	85.02 (±12.8)	87.58 (±10.2)
p-value	0.55	0.016	0.36	0.56	0.025	0.045
Distance from CI centre						
Less than 200 km	90.87 (±11.7)	83.65 (±17.7)	83.5 (±17.3)	78.82 (±15.6)	80.87 (±15)	84.14 (±12.1)
More than 200 km	95.12 (±9.3)	90.97 (±12)	91.25 (±11.2)	82.56 (±18)	87.87 (±11.2)	90.1 (±9.1)
p-value	0.09	0.043	0.027	0.415	0.028	0.021
Experienced complications related to surgery						
Yes	93.65 (±8)	87.86 (±19.1)	90 (±14.4)	85 (±15.4)	88.34 (±13.1)	90.56 (±10.6)
No	92.9 (±11)	87.2 (±15.2)	87.05 (±15.2)	80.3 (±17)	83.9 (±13.7)	86.71 (±11.1)
p-value	0.86	0.92	0.62	0.56	0.41	0.30
Experienced technical problems related to CI						
Yes	94.44 (±6.5)	91.25 (±8.7)	86.88 (±14.6)	91.67 (±6.8)	89.69 (±6.3)	90.19 (±7.1)
No	92.8 (±11.1)	86.77 (±16.1)	87.38 (±15.2)	79.38 (±17.2)	83.67 (±14.2)	86.7 (±11.4)
p-value	0.68	0.44	0.93	0.09	0.24	0.41
Compliance to speech rehabilitation						
Yes	93.8 (±11.1)	87.66 (±15.8)	86.49 (±16)	81.34 (±15.7)	84.93 ( 13.6)	87.69 (±10.9)
No	91.47 (±10)	86.54 (±15.1)	88.85 (±13.2)	79.55 (±19)	83.24 (±13.9)	85.98 (±11.5)
p-value	0.38	0.77	0.52	0.71	0.62	0.53

**Table 5:** The correlation of the studied factors with PedsQL - GCS subscales and total scores among children with CI

Characteristics	Physical PedsQL	Emotional PedsQL	Social PedsQL	School PedsQL	Psychosocial PedsQL	Total PedsQL
Age (in months)						
p-value	0.089	0.769	0.345	0.304	0.9	0.72
Age at diagnosis (in months)						
p-value	0.308	0.507	0.421	0.427	0.32	0.203
Age at cochlear implantation (in months)						
p-value	0.184	0.33	0.925	0.658	0.452	0.227
Duration since CI (in months)						
p-value	0.952	0.362	0.795	0.103	0.658	0.489
IQ						
p-value	0.408	0.877	0.515	0.83	0.692	0.429

Females were found to have higher scores than males in emotional and psychosocial dimensions (p = 0.033 and 0.041, respectively). The birth order in the family was found to be a significant predictor of child emotional, psychosocial, and total PedsQL scores (p = 0.016, 0.025, and 0.045, respectively). ANOVA with post-hoc corrections was conducted to compare the results for the three groups. The middle-born children had significantly higher emotional, psychosocial, and total PedsQL scores than the first-born children (p = 0.012, 0.022, and 0,034, respectively). There were no other significant differences. Children who are living with more than 200 km distance from CI centre were found to have higher scores than those who are living within a distance of 200 km in emotional, social, psychosocial dimensions, and total PedsQL score (p = 0.043, 0.027, 0.028 and 0.021, respectively).

The mothers’ education level was initially found to be a significant predictor of child social PedsQL scores (p = 0.035). However, when post-hoc corrections were conducted to compare the results for each pair of the five groups, no significant differences were found. In addition, we noted that the five groups are not evenly distributed in our sample. Therefore, the data are not sufficient to make any conclusion and a larger study is needed to provide statistical evidence for this point. Other factors including age, fathers' educational level, family monthly household income, number of siblings, age at diagnosis, age of cochlear implantation, the duration of cochlear implantation, whether the child had experienced complications related to surgery, whether the child had experienced technical problems related to CI, compliance to speech rehabilitation, and the child’s IQ were not found to be associated with PedsQL - GCS subscales and total scores.

## Discussion

In recent years, researchers have started giving big attention to the concept of health and the development of multidimensional health-related QoL instruments. Also, they realized that these instruments should contain important items, such as physical, mental, and social health dimensions, as described by the WHO [7]. Inadequate management of hearing impairment can result in a delay to speech development, learning difficulties, poor academic performance, social isolation, low self-esteem, and subsequently poor QoL.

In our study, health-related QoL scores were measured using the PedsQL^TM^ 4.0 - GCS and we found that there were no significant differences between children with CI and normal children, in terms of physical, emotional, social, school, psychosocial, and total functioning, as the scores were comparable. Several studies were also done to compare the QoL in CI children and age-matched normal children and the majority were consistent with our findings [16,17]. It is generally known that CI has a positive impact on the recipients' speech and language development, and some aspects of psychosocial domains that are important for practicing regular daily activities, and thus, it leads to normal daily life for the recipients and their families [16,18]. In terms of age of cochlear implantation, it has been demonstrated that children who had earlier exposure to sounds (especially those who underwent implantation by 3.5 years of age) have better opportunities for age-appropriate auditory development, normal development of central auditory pathways, and as a result, they were more integrated and involved with the surroundings and less likely to feel isolated and left behind [16,17,19,20]. And while looking at our study sample, we determined that the mean age of cochlear implantation was at 2.7 years. 

The literature has suggested other different factors that can influence the auditory and speech outcomes, and the perceived QoL among CI children. These include compliance to speech rehabilitation and socioeconomic factors including family household income and level of parents' education [21-25]. In our study, gender, birth order, and distance from the CI centre were found to have different effects on the QoL. According to Chaplin and Aldao, girls tend to decrease displays of externalizing negative emotions (anger, contempt, and disgust) and increase displays of positive emotions (happiness and surprise), in comparison to boys at toddler/preschool and childhood age periods [26], and among CI children, it has been reported that implanted girls are more prosocial than boys [27,28]. Our finding is consistent with the previous studies, with respect to males and females, as females scored higher than males in emotional and psychosocial dimensions. Although some might argue that first-born children might get more attention than their siblings, middle-born children were found to have a higher emotional, psychosocial, and total PedsQL score compared to the first-born children. This might be explained by the fact that older siblings can enrich aspects of younger children’s language development through “overheard” conversations [29], and they also can contribute to younger children’s development of social skills because of increased opportunities for socialization and demonstration of prosocial behaviour between the siblings [30]. In the Kingdom of Saudi Arabia, CI centres are distributed in the main big cities, and we found that children who are living more than 200 km from CI centre have higher emotional, social, psychosocial, and total scores than those who are living within a 200 km distance, which is predictable as children living more than 200 km from CI centre are more likely to be living in small towns and villages where social bonding and interpersonal connection between families and neighbours are relatively stronger. 

Interestingly, socioeconomic factors including the parents’ education level and monthly household income did not have a significant association with the PedsQL - GCS subscales and total scores. Parental and family participation in the rehabilitation sessions can improve the child’s language outcomes. Through the guidance and demonstration of speech and language pathologists, parents can play a positive role in the development of their child, regardless of their level of education. Fortunately, income disparities between families have a limited impact on the health of Saudi’s hearing-impaired children. In Saudi Arabia, the public health services and access to public and special education come from the government and they are free-of-charge. Moreover, the disability-related costs including transportation for hospital visits, CI batteries, and repairs, and rehabilitation sessions are usually funded by the government. The government also provides monthly economic support to families having a child with special needs. 

Our recommendations for a better QoL in CI child

(1) For early detection of hearing loss: Implementing a strict national newborn hearing screening program, and public awareness programs to educate parents about the early manifestations of hearing loss, the benefits of early intervention, and what to do if they suspect that their child has hearing loss.

(2) For equal healthcare: Reducing the impact of economic disparities on the health of hearing-impaired children.

(3) For easier services and more efficient rehabilitation: Providing a free and online unified platform for CI children and their families, through which they can be easily targeted with different educational content and rehabilitative services.

(4) For better education and social development: Using a classroom FM system. Training more teachers to evaluate and improve the academic performance of CI children and to encourage the communicative interaction between CI children and normal children.

(5) For active involvement of parents: Addressing the parents’ concerns and needs, and encouraging their involvement in the learning process of their child.

(6) For a better experience: Giving the CI recipients the possibility to follow the latest technology. For example, CI with Bluetooth-compatibility feature, directional microphone systems, noise reduction systems, data logging feature, MRI-compatibility, dust, and water resistance.

## Conclusions

In our study, we aimed to assess the QoL in Saudi CI children compared to age-matched normal children. We used PedsQL^TM^ 4.0 - GCS to assess the QoL in both groups and no significant differences were noted between them. However, among CI children, gender, birth order, and distance from the CI centre were found to have different effects on the QoL dimensions.
